# Oral desensitization to milk: how to choose the starting dose!

**DOI:** 10.1111/j.1399-3038.2009.00917.x

**Published:** 2010-03

**Authors:** Francesca Mori, Neri Pucci, Maria Elisabetta Rossi, Maurizio de Martino, Chiara Azzari, Elio Novembre

**Affiliations:** Allergy and Clinical Immunology Unit, A. Meyer Children Hospital, Department of Pediatric, University of FlorenceFlorence, Italy

**Keywords:** children food allergy, end point skin prick test, milk allergy, oral desensitization

## Abstract

Mori F, Pucci N, Rossi ME, de Martino M, Azzari C, Novembre E. Oral desensitization to milk: how to choose the starting dose! Pediatr Allergy Immunol 2010: 21: e450–e453. © 2009 John Wiley & Sons A/S

A renewed interest in oral desensitization as treatment for food allergy has been observed in the last few years. We studied a novel method based on the end point skin prick test procedure to establish the starting dose for oral desensitization in a group of 30 children higly allergic to milk. The results (in terms of reactions to the first dose administered) were compared with a group of 20 children allergic to milk as well. Such control group started to swallow the same dose of 0.015 mg/ml of milk. None reacted to the first dose when administered according to the end point skin prick test. On the other side, ten out of 20 children (50%) from the control group showed mild allergic reactions to the first dose of milk. In conclusion the end point skin prick test procedure results safe and easy to be performed in each single child in order to find out the starting dose for oral desensitization to milk, also by taking into account the individual variability.

Nowadays several strategies are under investigation as new therapeutic approaches to treat food allergies: infusion of anti-IgE antibodies, bacterial agents, immunomodulatory agents, vaccination with plasmid DNA, chinese herbs, cytokines and cytokine modifiers, adhesion molecule antagonists, chemokine or chemokine receptor antagonists and oral desensitization (1). So far increasingly studies about oral desensitization, support the beneficial effects of this approach compared to avoidance of the allergenic food (2, 3). Oral desensitization has been performed with milk, eggs and peanuts showing that a forced tolerance is inducible but mostly dependent on a daily ingestion of the allergenic food (4, 5). Therefore oral tolerance should be limited to foods necessary for the normal growth and recommended just for widespread products easily disclosed as hidden allergens or as source of contamination underling a risk of life.

Different procedures suitable to induce cow’s milk oral tolerance have been recently suggested, but a well defined and standardized protocol for oral desensitization is still lacking (6–11).

We describe our experience with oral desensitization in children, delineating a new protocol based on the end point skin prick test procedure. This method has been resulted safe, reliable, easy and extremely useful to establish a single starting dose of allergen.

## Methods

### Subjects

We examined 50 children highly allergic to milk referred to a paediatric allergy centre. All children had a personal history of anaphylactic reaction to milk in the last 6 months - a year (12) or severe allergic reactions with very low doses of milk (<0.6 mg of cow’s milk proteins) (13). Personal history was taken by an allergologist and the severity of all clinical reactions described by patients was evaluated according to the current definitions of food allergy (14). None of them has been receiving antihistamines and systemic or topical corticosteroids during the 2 wk before clinical evaluation. Moreover all children were older than 3 yr of age in order to rule out the 87% of cases naturally outgrowing allergy/intolerance to milk (15). All patients were randomly split in two groups: 30 children followed our protocol based on the end point skin prick test and a control group of 20 children started to swallow the same dose of milk. The progressive milk intakes followed the same scheme for both groups.

Both parents of all children enrolled were required to sign an informed consent.

The study was approved by an ethics committee.

### End point skin prick testing method

In 30 children skin prick tests were performed with fresh cow’s milk (30 mg/ml) and its progressive dilutions (1/10 = 3 mg/ml; 1/100 = 0.3 mg/ml; 1/1000 = 0.03 mg/ml; 1/10,000 = 0.003 mg/ml; 1/100,000 = 0.0003 mg/ml; 1/1,000,000 = 0.00003 mg/ml). Dilutions were prepared under sterile conditions. We progressively diluted fresh cow’s milk with saline solution in 10 ml plastic tubes. For the dilution 1/10 we added 9 ml of saline solution to 1 ml of fresh milk. To obtain the dilution 1/100 we added 9 ml of saline solution to 1 ml drawn out from the 1 to 10 dilution and so on. In 20 children we performed skin prick tests only with fresh cow milk. We considered positive a wheal skin reaction ≥3 mm of diameter. We obviously performed a positive and a negative skin prick test as controls for each patient enrolled.

### Oral desensitization protocol

The first day, 30 children started with the dilution immediately below the end point one (positive to the skin prick tests) and we progressively increased every 20 min the amount of milk administered according to this scheme: 1 drop; 2 drops; 4 drops; 8 drops. The next day we administered 1-2-4-8 drops at the end point dilution and the day after we administered the dilution immediately above the end point dilution according to the same dosages and timing. Every day we increased the doses until we reached the pure milk. By this time even if the increments were mostly dependent on the severity of the allergic reactions occurred, we followed this scheme: 1 (=1.5 mg/ml of cow milk proteins) drop, 2, 4, 8 drops (every 20 min, the first day); 8, 16 drops, 1 ml (about 20 drops) (the second day). At this time point we administered 3–4 doses per day, doubling the amount at every step. In case of reactions, we stopped the procedure for that day and the day after we started from half the dose provoking allergic manifestations. The control group of 20 children started with 0.015 mg/ml of milk (1 drop of pure milk in 99 drops of water). The amount of 100 ml of milk was considered the ending dose and it was reached in approximately 6 months. Usually we performed the higher increments at the hospital, discharging the patient with half of the dosage reached. During oral desensitization children were completely free from any pre- or co-treatment with antihistamines or corticosteroids.

### Immunologic parameters, clinical diary and medical treatment recorded

At the beginning of oral desensitization blood samples were collected to determine specific serum IgE levels for both milk and cow’s milk proteins: casein, α-lactoalbumin and β-lactoglobulin (Radio-Allergo-Sorbent Test RAST).

All the first allergic reactions occurred into the hospital and a physician wrote them down in a case history. When necessary a therapy with antihistamines, corticosteroids and bronchodilators was performed according to the severity of the reactions. Only one patient required a treatment with adrenalin.

### Statistical analysis

All data were elaborated by using a commercially available statistical software package (SPSS, Chicago, IL, USA). Student’s *t-*tests for independent samples were used for the comparison of mean values. Probability values of less than 0.05 were considered statistically significant.

## Results

The age of the 30 children evaluated (21 males and 9 females) was 36–190 months [mean age (±s.d.) 81.8 ± 43.69 months[. The specific serum IgE mean (±s.d.) for cow’s milk was 33 ± 32.87 KUA/L (range 0.91–100 KUA/L). We found a significantly higher mean (±s.d.) of casein specific serum IgE levels (30.15 ± 34.96) compared to the means (±s.d.) of the two other serum cow’s milk proteins α-lactoalbumin and β-lactoglobulin (9.36 ± 14.43; 6.31 ± 12.19 p < 0.001 respectively).

**Fig. 1:**
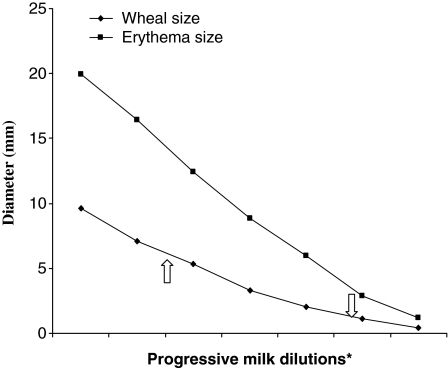
End point skin prick tests results in relation to progressive milk dilutions (*from pure milk to 1/1,000,000 dilution). 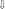 wheal mean size corresponding to the starting dose administered for oral desensitization (1.42 ± 0.8 mm). 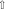 wheal mean size corresponding to the first dilution provoking allergic reactions (6.64 ± 4.069 mm).

The distributions of both the wheal and the flares size diameters obtained from the skin prick tests performed with progressive dilutions (according to the end point procedure) by starting from pure cow’s milk were following a fall line (Fig. 1).

The end point skin prick test dilutions of cow’s milk obtained were: 2 (6.7%) patients had the threshold concentration at 1:100,000; 10 (33.3%) at 1:10,000; 13 (43.3%) at 1:1000; 4 (13.3%) at 1:100 and 1 (3.3%) at 1:10.

We calculated the wheal mean size corresponding to the starting dose administered for oral desensitization (1.42 ± 0.8 mm) and we compared the value obtained to the wheal mean size corresponding to the first dilution provoking allergic reactions (6.64 ± 4.069 mm p < 0.001). Two patients had never showed reactions during desensitization.

None had reaction to the starting dose administered according to the end point skin tests results (0%). In addition, 3 (10.71%) children had never showed allergic reactions until large doses (>50 ml) of pure milk were reached; 11 (39.28%) had allergic reactions as soon as pure milk was administered**;** 7 (25%) had reactions to the end point dilution and 7 (25%) had reactions between the dilution immediately above the end point one and the pure milk intake.

As reported in the table below (Table 1) all the first reactions were mild allergic reactions (16) with mostly a skin involvement.

**Table 1:** Early allergic reactions occurred during oral desensitization

Clinical allergic reactions	N (%)
Skin reactions (urticaria)	13 (35.2)
Oral Allergic Sindrome (OAS)	9 (24.3)
Gastrointestinal symptoms	6 (16.2)
Rhinitis	3 (8.1)
Cough	3 (8.1)
Congiuntivitis	1 (2.7)
Wheezing	1 (2.7)
Angioedema	1 (2.7)
Total	37 (100)

Patients from the control group were matched with the 30 children for age and milk specific serum IgE levels. Ten out of 20 children (50%) from the control group showed mild allergic reactions with the starting dose administered. Comparing the percentages of children reacting to the first dose of milk in both the two groups (0% vs. 50%), we can asses that the end point procedure allow us to be more confident with each single child, reducing the risk of reaction at the beginning. Despite mean values are used, all results reported have a normal distribution (data not shown).

## Discussion

Oral desensitization is still an attracting procedure although it is known since 1984 (17). New approaching protocols have been proposed in the past few years, but no consensus has been reached on the starting doses and on the timing necessary to induce tolerance. Moreover none reported the dose provoking the first clinical reaction during oral desensitization.

Our results show the safety of this new protocol to start oral desensitization to milk. None reacted to the first dilution administered even if none was pre or cotreated with anti-allergic drugs.

Our proposal is based on the observation that serum specific IgE levels, triggering dose of allergenic food and severity of reactions seem to greatly vary even into the same group of termed highly allergic children.

Personalizing the starting dose according to the self allergic skin reaction has been resulted safe in 30 children referred to our hospital. Moreover avoiding antihistamines before or during oral desensitization, as other authors reported, makes clear the evaluation of the clinical reactions to the allergen and it gives us the possibility to slow down or go head with more reliability during the procedure.

As published for oral challenge a wheal size >7–8 mm for milk, peanuts and soy has a positive predictive value of 90% to have an allergic reaction (18). Our findings for highly allergic children showed a skin prick tests value of 6.64 mm as threshold wheal size related to an allergic reaction. Some more studies had been considering the correlation between serum specific IgE levels and the probability to have positive reactions during a challenge with the allergenic food under investigation (16). We are still following the fifty children by measuring specific serum IgE levels and some preliminary results show their progressive decrement during desensitization in agreement with previous studies (19).

In conclusion our procedure allows to be more confident with each single child when starting oral desensitization and it offered a better standardized approach to force tolerance avoiding individual variability.
